# Structural and biochemical analysis of a thermostable membrane-bound stomatin-specific protease

**DOI:** 10.1107/S0909049513021328

**Published:** 2013-09-25

**Authors:** Hideshi Yokoyama, Daisuke Kobayashi, Naoto Takizawa, Satoshi Fujii, Ikuo Matsui

**Affiliations:** a School of Pharmaceutical Sciences, University of Shizuoka, 52-1 Yada, Suruga-ku, Shizuoka 422-8526, Japan; b Biomedical Research Institute, National Institute of Advanced Industrial Science and Technology (AIST), 1-1 Higashi, Tsukuba, Ibaraki 305-8566, Japan

**Keywords:** membrane-bound protease, stomatin, thermostable, substrate peptide, *Pyrococcus horikoshii*

## Abstract

Membrane-bound proteases are involved in various regulatory functions. The N-terminal region of PH1510p (1510-N) from the hyperthermophilic archaeon *Pyrococcus horikoshii* is a serine protease with a catalytic Ser–Lys dyad (Ser97 and Lys138), and specifically cleaves the C-terminal hydrophobic region of the p-stomatin PH1511p. In a form of human hemolytic anemia known as hereditary stomatocytosis, the stomatin protein is deficient in the erythrocyte membrane due to mis-trafficking. In order to understand the catalytic mechanism of 1510-N in more detail, here the structural and biochemical analysis of 1510-N is reported. Two degraded products were produced *via* acyl-enzyme intermediates. 1510-N is a thermostable protease, and thus crystallization after heat treatment of the protease–peptide complex was attempted in order to understand the catalytic mechanism of 1510-N. The structure after heat treatment is almost identical to that with no heat treatment. According to the superposition between the structures with heat treatment and with no heat treatment, the N-terminal half of the peptide is superposed well, whereas the C-terminal half of the peptide is slightly deviated. The N-terminal half of the peptide binds to 1510-N more tightly than the C-terminal half of the peptide. The flexible L2 loops of 1510-N cover the peptide, and are involved in the protease activity.

## Abstract 2

According to the structural and biochemical analysis of the stomatin-specific protease 1510-N, two degraded products were produced *via* acyl-enzyme intermediates. The N-terminal half of the substrate peptide binds to 1510-N more tightly than the C-terminal half of the peptide.

## 1. Introduction   

Membrane-bound proteases play several important roles in protein quality control and regulation (Brown *et al.*, 2000 ▶). Several archaeal proteases were investigated by functional analyses such as Lon protease (Fukui *et al.*, 2002 ▶) and signal peptide peptidase (Matsumi *et al.*, 2006 ▶) from *Thermococcus kodakaraensis* KOD1. The hyperthermophilic archaeon *Pyrococcus horikoshii* grows optimally at about 373 K, and its highly thermostable gene products are good candidates for the functional and structural analyses.

Stomatin, prohibitin, flotillin and HflK/C (SPFH) domain proteins are found in lipid raft microdomains in various cellular membranes (Tavernarakis *et al.*, 1999 ▶; Browman *et al.*, 2007 ▶). Human stomatin has been identified as a major component of vesicles produced by red cells (Salzer *et al.*, 2008 ▶). In a form of hemolytic anemia known as hereditary stomatocytosis, the stomatin protein is deficient in the erythrocyte membrane (Stewart *et al.*, 1993 ▶). Stomatin is mis-trafficked in the erythrocytes of hereditary stomatocytosis (Fricke *et al.*, 2005 ▶), and the conditions are not caused by mutations in the stomatin gene but by mutations in Rh-associated glycoprotein (RHAG) (Bruce *et al.*, 2009 ▶) and glucose transporter 1 (GLUT1) (Flatt *et al.*, 2011 ▶). We determined the first crystal structure of the p-stomatin (prokaryotic stomatin) PH1511p from *P. horikoshii*. In the structure, the SPFH domain forms a stable trimer, and three C-terminal α-helical domains extend from the apexes of the triangle (Yokoyama *et al.*, 2008 ▶). In the first crystal structure of the mouse stomatin as eukaryotes, the SPFH domain assembles into a banana-shaped dimer (Brand *et al.*, 2012 ▶). Both structures determined from *P. horikoshii* and mouse do not contain membrane-spanning regions.

Stomatin-like proteins are found in almost all species of eukaryotes, bacteria and archaea (Tavernarakis *et al.*, 1999 ▶). In both archaeal and bacterial species, p-stomatin and STOPP (stomatin operon partner protein) genes probably form an operon (Green *et al.*, 2004 ▶). In *P. horikoshii*, there are two sets of STOPP/stomatin gene pairs, PH1510/PH1511 and PH0471/PH0470. The N-terminal region of STOPP PH1510p (residues 16–236, 1510-N) is a thermostable serine protease with a catalytic Ser–Lys dyad (Ser97 and Lys138), and specifically cleaves the C-terminal hydrophobic region of the p-stomatin PH1511p (^235^VIVL↓MLPM^242^ in which the arrow indicates the point cleaved) (Yokoyama & Matsui, 2005 ▶). We made the catalytically inactive mutants S97A and K138A, both of which show no activity (Yokoyama & Matsui, 2005 ▶; Yokoyama *et al.*, 2006 ▶), and determined the crystal structure of the K138A mutant of 1510-N in complex with a 10-amino-acid peptide of the p-stomatin PH1511p (Yokoyama *et al.*, 2012 ▶). In the structure, a 1510-N dimer binds to one peptide. The pseudo-two-fold axis running between two protomers of the 1510-N dimer also runs through the peptide bond between Val237 and Leu238 of the peptide. And the central six residues (^235^VIVLML^240^) of the peptide are hydrophobic and in a pseudo-palindromic structure, and therefore favorably fit into the hydrophobic active tunnel of the 1510-N dimer, although 1510-N degrades the substrate at only one point.

In order to understand the catalytic mechanism of 1510-N in more detail, we attempted to crystallize the catalytically inactive mutants S97A and K138A of 1510-N in complex with three kinds of substrate peptides. We also attempted crystallization of heat-treated protein–peptide complexes. Here we report the structural and biochemical analysis of 1510-N.

## 2. Materials and methods   

### 2.1. Biochemical analysis of 1510-N   

The wild-type 1510-N and 1511-C (residues 189–266 of p-stomatin PH1511p) were prepared as described previously (Yokoyama & Matsui, 2005 ▶; Yokoyama *et al.*, 2006 ▶). For the preparation of other substrates, the DNA fragments encoding residues 168–266, 189–256 and 168–256 of PH1511 were amplified by PCR using the expression vector for PH1511 as a template, and the resultant vectors were prepared. Expression and purification of the substrate proteins were carried out in the same protocol as described by Yokoyama & Matsui (2005 ▶). For the preparation of an L2-deletion mutant of 1510-N (1510-N Δ126–133), the expression vector for residues 16–125 and 134–236 of PH1510p were prepared. Protein expression and purification were carried out in the same protocol as described previously (Yokoyama & Matsui, 2005 ▶; Yokoyama *et al.*, 2012 ▶). All the resultant proteins described above additionally contain initial methionine at their N-termini and LEHHHHHH at their C-termini. The wild-type or L2-deletion mutant of 1510-N was mixed with the substrate in MES buffer (pH 6.0), and incubated at 353 K. SDS-PAGE of the reaction mixture was performed.

### 2.2. Crystallization   

The catalytically inactive mutants S97A and K138A of 1510-N were prepared as described previously (Yokoyama & Matsui, 2005 ▶; Yokoyama *et al.*, 2012 ▶). As substrates, three kinds of synthetic peptides, ^234^NVIVLMLPME^243^, ^232^KSNVIVLML^240^ and ^238^LMLPMEMLK^246^ (superscripts show residue numbers of p-stomatin PH1511p) were used, and referred to as 234P10, 232P9 and 238P9 peptides, respectively. The purified inactive mutants were mixed with one of the synthetic peptides at a molar ratio of 1:10, and crystallization trials were performed. Crystallization drops were prepared by mixing equal volumes of the protease–peptide and reservoir solutions. For crystallization of the heat-treated protease–peptide complex, the protease–peptide complex was heated at 353 K for 10 min, kept on ice for 10 min, and then crystallization trials were performed.

The protein–peptide solution contained 5.2 mg ml^−1^ of 1510-N K138A and 2.4 mg ml^−1^ of the 234P10 peptide in a buffer containing 40 m*M* Tris-HCl (pH 8.5), 0.15 M NaCl and 4.8% (*v*/*v*) dimethylsulfoxide. Crystals obtained by heat treatment were grown at 293 K with the hanging-drop vapor-diffusion method, using a reservoir solution containing 0.8 *M* imidazole (pH 7.5). Cubic crystals grew to an approximate size of 0.15 mm per side.

### 2.3. Data collection and structure determination   

The crystal was cryoprotected in a solution containing 1.0 *M* imidazole (pH 7.5), 30% (*v*/*v*) glycerol, and flash-frozen at 100 K. X-ray diffraction data were collected at beamline BL26B1 of SPring-8 (Hyogo, Japan) with a Saturn A200 CCD detector, and processed and scaled with *HKL2000* (Otwinowski & Minor, 1997 ▶).

The structure was determined by the TLS-restrained crystallographic refinement with *REFMAC5* (Winn *et al.*, 2001 ▶) in the CCP4 suite (Winn *et al.*, 2011 ▶) using the structure of 1510-N K138A in complex with the 234P10 peptide (PDB code, 3viv) as the initial model by slight model fitting with *COOT* (Emsley & Cowtan, 2004 ▶). The least-squares fitting between two structures was performed with *LSQKAB* in the CCP4 suite. All molecular figures were produced with *PyMOL* (http://www.pymol.org/). The atomic coordinates and structure factors have been deposited in the RCSB Protein Data Bank with the accession code 3wg5.

## 3. Results and discussion   

### 3.1. Two degraded products are produced *via* putative acyl-enzyme intermediates   

**Figure 1:**
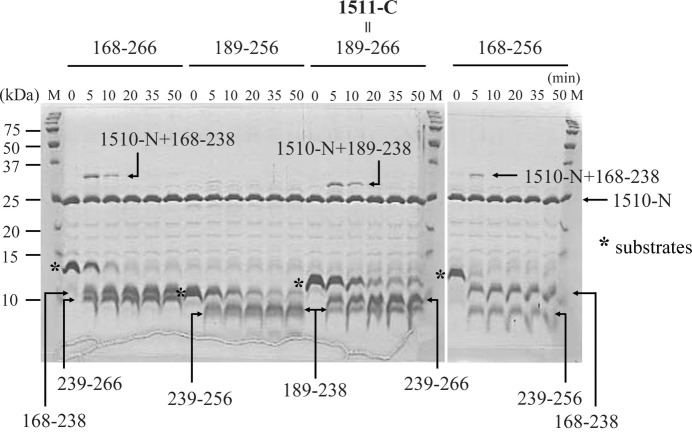
SDS-PAGE of protease–peptide reaction mixtures. Each substrate of residues 168–266, 189–256, 189–266 or 168–256 of PH1511p (1 µg) was degraded by the 1510-N protease (0.25 µg) with incubation at 353 K in a buffer containing 50 m*M* MES-NaOH (pH 6.0). Protease, substrate and degraded products after 0, 5, 10, 20, 35 and 50 min reaction were observed. Asterisks indicate substrate bands. Two degraded products were definitely identified as indicated by residue numbers. Putative acyl-enzyme intermediates were also detected as indicated. Lane M indicates molecular markers.

The 1510-N protease degrades the substrate 1511-C as shown in Fig. 1 ▶. According to the N-terminal sequence and LC-ESI-MS analyses, the upper product corresponds to the residues 239–266 of PH1511p, and the lower product corresponds to the residues 189–238 of PH1511p (Yokoyama *et al.*, 2012 ▶). These results indicated that 1510-N degrades the substrate 1511-C between Leu238 and Met239 at only one point. As we prepared several constructs of PH1511p for crystallization trials, protease reactions of 1510-N were performed against the substrates. Two degraded products were detected in each case using four kinds of substrates, and the two products were definitely identified as shown in Fig. 1 ▶. The degraded product 239–266 band is located above the product 189–238 band. At the C-terminus of PH1511p (266 residues), successively charged residues are present (^260^KKKEEEK^266^). Therefore, the degraded product 239–266 shows unusual mobility by SDS-PAGE. Interestingly, extra bands located above the 1510-N band were observed. The molecular masses of the extra bands correspond to acyl-enzyme intermediates. In the case of typical serine proteases, the serine hydroxyl group attacks the carbonyl group of the amide bond within the protein substrate, and the acyl-enzyme intermediate is formed (the covalent bond between the N-terminal product and protease is formed). Then an activated water molecule acts as the nucleophile, and the N-terminal product is released from the protease (Paetzel & Dalbey, 1997 ▶). The intermediates produced from the substrates 168–266 and 168–256 are almost the same size, and these intermediates are larger than the intermediate produced from the substrate 189–266. These results strongly indicate that the extra bands correspond to acyl-enzyme intermediates.

### 3.2. The N-terminal half of the substrate peptide binds to 1510-N more tightly than the C-terminal half   

In the previously determined structure of 1510-N K138A in complex with the 234P10 peptide (^234^NVIVLMLPME^243^), the 8-aa peptide ^234^NVIVLMLP^241^ is modeled (Yokoyama *et al.*, 2012 ▶). The catalytic Ser97 Oγ of chain A is hydrogen-bonded to the Asn234 O of the peptide, and the catalytic Ser97 Oγ of chain B is hydrogen-bonded to the Leu240 O. The structure shows the first substrate-binding step of 1510-N. 1510-N degrades the substrate 1511-C between Leu238 and Met239 at only one point (Yokoyama & Matsui, 2005 ▶). Thus, Ser97 of chain A should move along the substrate 4-residue C-terminally, or Ser97 of chain B should move along the substrate 2-residue N-terminally. In order to elucidate the catalytic mechanism of the 1510-N protease, we attempted to crystallize another protease–peptide complex using the substrates 232P9 (^232^KSNVIVLML^240^) and 238P9 (^238^LMLPMEMLK^246^). Until now, however, only the complex of 1510-N K138A and 234P10 peptide (^234^NVIVLMLPME^243^) was successfully crystallized.

1510-N is a thermostable protease, and the elevated activity was observed with temperatures from 323 to 371 K (Yokoyama & Matsui, 2005 ▶). Thus, we presume that heat treatment is a good candidate for approaching the second catalytic step of 1510-N. The protein–peptide complex was heated at 353 K for 10 min, and then crystallization trials were performed. Using the heat-treatment complex of 1510-N K138A and 234P10 peptide, crystals were obtained under the condition similar to that of the no heat-treatment complex obtained previously (Yokoyama *et al.*, 2012 ▶), and the structure was determined (Table 1 ▶).

**Figure 2:**
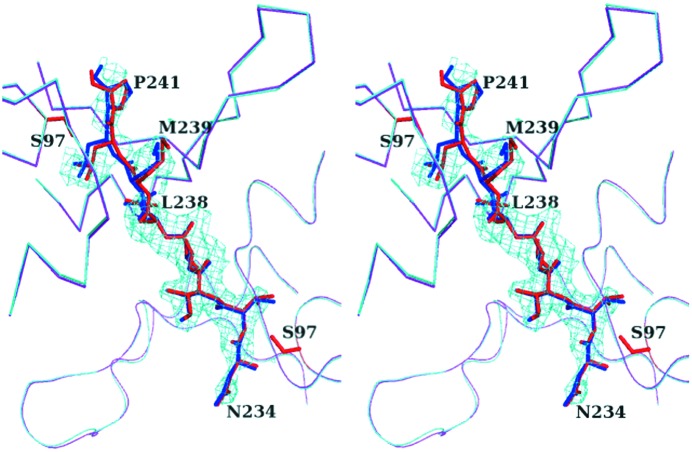
Superposition of the structures with heat treatment and with no heat-treatment of 1510-N K138A–234P10, and *F*_o_ − *F*_c_ electron densities of the heat-treatment complex (in a stereoview). Chains A and B are shown as cartoon loops and Cα traces, respectively. The peptide is shown as stick models. The complex with heat treatment is colored magenta (chains A and B) and red (peptide), and that with no heat treatment is colored cyan (chains A and B) and blue (peptide). The catalytic Ser97 residues of the heat-treatment complex are shown as red sticks. The electron densities of the peptide (residues 234–241) of the heat-treatment complex were calculated with phases from the model without the peptide.

**Figure 3:**
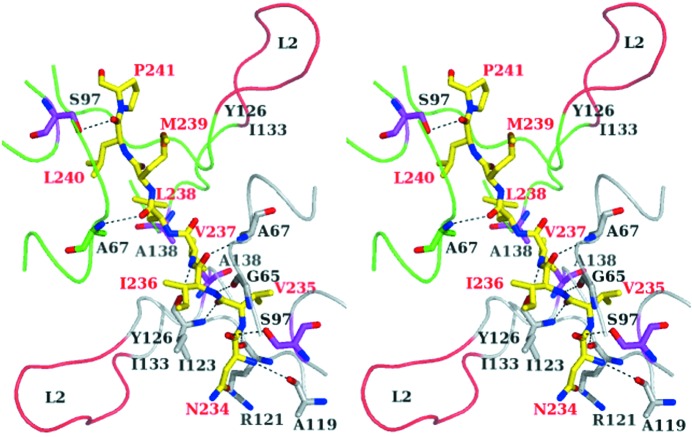
The peptide binding site of the heat-treatment complex of 1510-N K138A–234P10 (in a stereoview). One monomer (chain A) of 1510-N K138A is colored grey, and the other monomer (chain B) of 1510-N K138A is colored green. The peptide is shown as a yellow stick. The catalytic Ser97 and Ala138 (replaced Lys) residues are shown as magenta sticks. The tips of L2 loops (residues 126–133) are colored red. The view is almost the same as in Fig. 2 ▶.

The structure with heat treatment determined here is fitted to the structure with no heat-treatment determined previously (Yokoyama *et al.*, 2012 ▶), resulting in a low root-mean-square (r.m.s.) difference of 0.15 Å for Cα atoms of the 1510-N dimer, and 0.40 Å for all atoms of the peptide (Fig. 2 ▶). The result indicates that the structure with heat treatment is almost identical to that with no heat-treatment. The N-terminal half of the peptide (^234^NVIVL^238^) shows clear electron densities, whereas the C-terminal half of the peptide (^239^MLP^241^) shows weak densities (Fig. 2 ▶). Almost all the main-chain nitrogen and carboxyl oxygen atoms of ^234^NVIVL^238^ are hydrogen-bonded to the protease. The main-chain atoms of ^239^MLP^241^ have fewer hydrogen bonds than ^234^NVIVL^238^ (Fig. 3 ▶). These features are also observed in the structure of no heat-treatment. According to the superposition between the structures with heat-treatment and with no heat-treatment, the ^234^NVIVL^238^ peptide is superposed well, whereas the ^239^MLP^241^ peptide is slightly deviated (Fig. 2 ▶). The distance between both Pro241 Cα atoms of the two structures is 0.74 Å. According to the results of hydrogen bonds and superposition, the N-terminal half of the peptide binds to 1510-N more tightly than the C-terminal half of the peptide.

### 3.3. Flexible L2 loops are involved in the protease activity   

**Figure 4:**
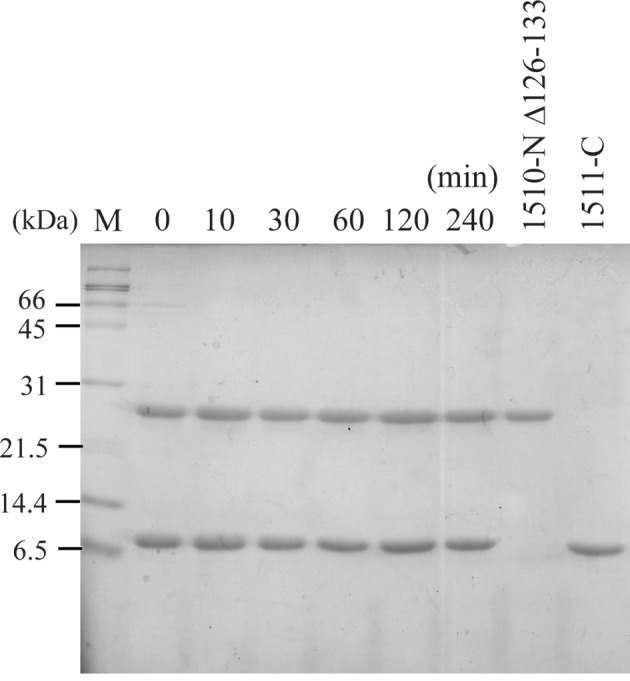
SDS-PAGE of the incubation mixture of 1510-N Δ126–133 and 1511-C. 1510-N Δ126–133 (1 µg) was mixed with the substrate 1511-C (2.5 µg) in 20 m*M* MES-NaOH (pH 6.0), and incubated at 353 K for 0, 10, 30, 60, 120 and 240 min. Products degraded by 1510-N Δ126–133 were not detected. Lane M indicates molecular markers.

The two long L2 loops of the 1510-N dimer cover the peptide (Fig. 3 ▶). In order to understand how the L2 loops of 1510-N contribute to the protease activity, we made an L2-deletion mutant of 1510-N (1510-N Δ126–133). 1510-N Δ126–133 and the substrate 1511-C were incubated at 353 K. Even in the long incubation using a large amount of the protease, products degraded by 1510-N Δ126–133 were not detected (Fig. 4 ▶). This result indicates that the L2 loop affects the protease activity.

One of the catalytic residues Lys138 is located at the base of the L2 loop. The two catalytic Ala138 residues that replaced Lys are located very close together (Fig. 3 ▶). Thus in the wild-type 1510-N, the close positioning of the catalytic Ser97 and Lys138 may be induced by electrostatic repulsion of the two Lys138 side-chains of the protomers. Unfortunately, we could not obtain the structure corresponding to the second catalytic step of 1510-N. If we can stably obtain the acyl-enzyme intermediate using the mutant 1510-N, we might elucidate the second catalytic step triggered by the conformational change of Lys138.

## Supplementary Material

PDB reference: 3wg5

## Figures and Tables

**Table 1:** Data collection and refinement statistics Values in parentheses are for the highest-resolution shell.

Data collection	
Space group	*P*4_3_2_1_2
Cell dimensions ()	*a* = 111.4, *c* = 91.7
Wavelength ()	1.0000
Resolution range ()	202.40 (2.442.40)
No. of observed reflections	230168
No. of unique reflections	23169 (1136)
*R*_merge_ (*I*)†	0.049 (0.325)
Completeness	0.999 (1.000)
Average *I*/	61.9 (7.8)

Refinement	
Resolution range ()	202.40
No. of reflections used	20752
Completeness (%)	0.996
*R*_work_ ‡/*R*_free_ §	0.202/0.241
No. of non-hydrogen atoms	
Protein	3374
Peptide	61
Solvent	168
Average *B* factors (^2^)	
Protein	56.6
Peptide	88.1
Solvent	58.8
R.m.s. deviations from ideality	
Bond lengths ()	0.009
Bond angles ()	1.171
Ramachandran plot¶ (%)	
Favored region	97.0
Allowed region	3.0
Outlier region	0

† *R*_merge_(*I*) = _*hkl*__*j*_|*I*_*j*_(*hkl*) *I*(*hkl*)|/_*hkl*__*j*_ *I*_*j*_(*hkl*), where *I*_*j*_(*hkl*) is the intensity of an individual reflection and *I*(*hkl*) is the mean intensity of that reflection.

‡ *R*_work_ = _*hkl*_||*F*_obs_| |*F*_calc_||/_*hkl*_|*F*_obs_|, where |*F*_obs_| and |*F*_calc_| are the observed and calculated structure factor amplitudes for working-set reflections, respectively.

§ *R*_free_ is calculated for 10% of the reflections randomly excluded from refinement.

¶ Values for proteins and the peptide were calculated with *RAMPAGE* (Lovell *et al.*, 2003 ▶).
